# Effect of Pretreatment and Drying on the Nutritional and Functional Quality of Raisins Produced with Seedless Purple Grapes

**DOI:** 10.3390/foods13081138

**Published:** 2024-04-09

**Authors:** Fengjuan Liu, Jing Lei, Xupeng Shao, Yingying Fan, Wei Huang, Weijia Lian, Tao Sun, Ya Chen, Cheng Wang

**Affiliations:** 1 Laboratory of Quality and Safety Risk Assessment for Agri-Products (Urumqi), Key Laboratory of Functional Nutrition and Health of Characteristic Agricultural Products in Desert Oasis Ecological Region (Co-Construction by Ministry and Province), Ministry of Agriculture and Rural Affairs, Institute of Quality Standards & Testing Technology for Agri-Products, Xinjiang Academy of Agricultural Sciences, Urumqi 830091, China; liufengjuan@xaas.ac.cn (F.L.); shaoxupeng@xaas.ac.cn (X.S.); fyyxaas@xaas.ac.cn (Y.F.); suntao26@xaas.ac.cn (T.S.); 2 Turpan Institute of Agricultural Science, Xinjiang Academy of Agricultural Science, Turpan 838000, China; tlfleijing@126.com (J.L.); lwj1634321773@126.com (W.L.); chenya2024@126.com (Y.C.); 3 Microbial Application Institute, Xinjiang Academy of Agricultural Sciences, Urumqi 830091, China; ryan_huangwei1991@126.com

**Keywords:** seedless purple raisins, pretreatment, nutritional quality, phenolic content, antioxidant activity

## Abstract

Raisins, known for their delicious taste and high nutritional value, are among the most widely consumed dried fruits globally. The natural waxy layer on the surface of grapes impedes water migration, making pretreatment necessary before drying. This study evaluated the effects of various pretreatment methods on the nutritional and functional quality of seedless purple raisins. By using non-pretreated dry seedless purple raisins as a control, the impact of physical and chemical pretreatment methods on the nutritional and functional qualities of seedless purple raisins was assessed through the analysis of nutrient content, phenolic compounds, and antioxidant activity. Our results demonstrate that physical pretreatment significantly increases the levels of vitamin C, fructose, glucose, total acid, total phenolics, total flavonoids, total anthocyanins, and antioxidant activity compared to chemical pretreatment and the control group. The correlation analysis revealed that phenolic substances were closely linked to antioxidant capacity. Additionally, phenolic compounds, including resveratrol, ferulic acid, chlorogenic acid, ethyl coumarate, and cinnamic acid, were more abundant following physical pretreatment. The OPLS-DA model effectively differentiated the three groups of processed samples, showing that different pretreatments significantly affect the nutritional and functional quality of seedless purple raisins. These findings suggest that physical pretreatment offers considerable potential for improving the drying quality of seedless purple raisins.

## 1. Introduction

Grapes (*Vitis* sp.) are among the world’s most crucial horticultural crops, and are notable for their rich content of phytochemicals such as flavonoids, anthocyanins, tannins, phenolic acids, and minerals [1]. Grapes can be consumed in various forms, including fresh fruit, raisins, jams, juices, and wine, with drying serving as the main processing technique [2]. Raisins, recognized for their delightful taste and substantial nutritional value, rank as one of the most significant and favored dried fruits globally [3]. China stands as the world’s third-largest raisin producer, with Xinjiang Province leading in raisin production [4]. At present, common drying methods include shade drying, sun drying, and hot-air drying [5]. The shade drying of grapes takes about 30 days, while sun drying usually requires 8–10 days [6]. In contrast, hot-air drying significantly reduces the drying period to merely 2–3 days [7].

In transforming grapes into raisins, various intrinsic grape characteristics, such as the berry size, volume, sugar concentration, and the presence of a waxy membrane covering the cuticular epidermis, influence the drying rate. This waxy layer obstructs water loss, thus decelerating the dehydration process [7]. To accelerate drying, numerous chemical and physical pretreatments are utilized to improve permeability. Nonetheless, these pretreatments can impact product quality. For example, olive oil pretreatment preserves higher levels of anthocyanins and proanthocyanidins in raisins compared to untreated ones. The carbonic maceration (CM) technique has been shown to significantly increase drying rates and product quality [8], while NaOH pretreatment tends to decrease antioxidant activity [9]. Research has indicated that cold plasma pretreatment notably reduces the drying time and improves the quality of dried grapes, increasing the total phenolic content, antioxidant activity, and vitamin C, with a 50 s exposure being optimal for energy efficiency and a minimal impact on the color [5]. Air plasma treatment yields raisins with an appearance, color, and texture similar to that of untreated and chemically treated controls, yet with a more than twofold increase in the total phenolic content and antioxidant capacity [10]. Despite these advancements, some pretreatments that eliminate the waxy membrane can leave chemical residues, raising food safety issues, and produce wastewater containing corrosive, saline, and organic solids [11], leading to extra costs for producers [12]. Furthermore, with the growing demand for organic foods, the use of chemical additives is increasingly limited [13]. Hence, we have developed a grape pretreatment device that uses a physical friction drum to remove the wax from the grape surface, achieving pretreatment goals without using drying agents. This method is novel, scarcely reported in the literature, and offers the benefits of simplicity, convenience, and no residue.

China is renowned for its diverse grape varieties, among which seedless purple raisins are especially popular. The objective of this study was to assess the impact of physical pretreatment on seedless purple raisins in comparison to two conventional methods: treatment with drying agents and without any treatment. Raisins were produced through hot-air drying, and subsequent analyses were performed to profile their nutrient content and antioxidant properties. This comparative evaluation of pretreatments aims to provide empirical data supporting further research into the dry processing of seedless purple grapes. Additionally, it reinforces the case for using non-toxic and harmless physical pretreatment techniques in raisin production.

## 2. Materials and Methods

### 2.1. Plant Material

Seedless purple grapes (*Vitis vinifera* Black Monukka) were harvested from a garden in the city of Turpan (Xinjiang, China) located at 42°57′26″ N and 90°21′20″ E. The grapes were harvested at commercial maturity, with a Brix range of 20% to 22%. The selection criteria for the experiment were a uniform berry size (diameter: 12.51 mm ± 0.81 mm, length: 16.99 mm ± 1.28 mm), consistent coloration, and no surface damage or disease. The initial moisture content of the grapes was 79.14 ± 0.16% (wet basis).

### 2.2. Processing

**Figure 1:**
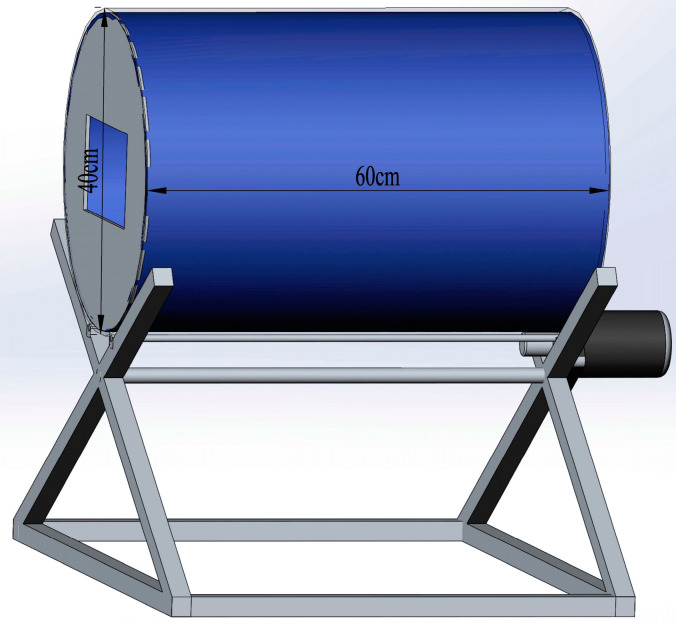
The motorized rotating drum used for the physical pretreatment of grapes, lined internally with sandpaper to remove the surface wax.

The seedless purple grapes were divided equally into three groups for pretreatment:

(1) In the physical treatment group (PT), based on preliminary experiments, the following conditions were applied: the grape surface wax was removed using a motorized rotating drum (D = 40 cm, L = 60 cm) lined with 500-grit sandpaper (Figure 1 for details). The drum rotated at 10 rpm, and the grapes underwent pretreatment for 10 min with a batch mass of 5 kg.
(2) In the drying agent treatment group (DT), the grape samples were immersed in a solution containing a 2.3% grape drying-promoting agent (Xinjiang HP Horticultural Technology Co., Ltd., Urumqi, China), composed of carbonate, lipid, and emulsifier components, for one minute.
(3) In the control group (CK), the grape samples received no pretreatment and were processed as is for comparative analysis.

The grapes from all three groups were subjected to hot-air drying, where 1000 g of grapes from each group was placed in a hot-air drying oven (DHG101S, Shaoxing Bowei Instrument Equipment Co., Ltd., Shaoxing, China) with temperature and air velocity controls (50 °C, 1 m/s). The drying process was monitored by weighing the samples every 2 h with an analytical balance until an 80% reduction in grape weight was achieved, corresponding to a moisture content of less than 15%. The samples from the PT required 38 h to dry, those from the DT took 54 h, and the CK samples needed 74 h to reach the desired endpoint.

### 2.3. Nutritional Quality

The total acidity was measured by titration with 0.1 N NaOH, expressed as grams of tartaric acid per kilogram of sample [14]. The fructose and glucose concentrations were analyzed after extraction using high-performance liquid chromatography (HPLC) (E2695, Waters Corporation, Milford, MA, USA) with a differential refraction detector, employing the external standard method (Figures S1 and S2 in Supplementary Materials). The procedure was as follows: 1.0 g of grape pulp was mixed with 50 mL of water, then 5 mL of zinc acetate solution and 5 mL of potassium ferrocyanide solution were gradually added. After thoroughly shaking the solutions, they underwent ultrasonic treatment for 30 min. The solution was filtered through filter paper, then further filtered through a 0.45 µm aqueous membrane syringe filter into the sample bottle for HPLC analysis. The chromatographic column used was an amino chromatography column (5 µm, 4.6 mm × 250 mm, MACHEREY-NAGEL, Wiesbaden, Germany), with a mobile phase of acetonitrile/water of 70:30 (*v*/*v*), a flow rate of 1 mL/min, a column temperature of 40 °C, and an injection volume of 10 µL. Amino acids were quantified after acid hydrolysis using a Hitachi L-8900 amino acid analyzer (Hitachi, Ltd., Tokyo, Japan) [15]. Specifically, in a vacuum environment, 2 g of grape pulp was hydrolyzed with 6 mol/L hydrochloric acid for 22 h at a hydrolysis temperature of 110 °C. After solution filtration, it was subjected to vacuum drying (RV8, IKA Works GmbH & Co., Staufenim Breisgau, Germany). The residue was dissolved in 1 mL of sodium citrate solution, and the filtrate was filtered through a 0.22 µm filter membrane before analysis. The column was a xanthate cationic resin column (7 µm, 4.6 mm × 150 mm, Sykam Scientific Instruments Co., Ltd., Beijing, China), with the detection wavelengths of the samples set at 570 nm and 440 nm (Figures S3 and S4 in Supplementary Materials).

### 2.4. Extraction of Phenolic Content

Phenolic extracts were obtained from the grapes as follows: the raisins were homogenized in acidified aqueous methanol (1 mol/L HCl: methanol: water, 1:80:19, *v*/*v*/*v*), which was followed by ultrasound-assisted extraction for 30 min at 25 °C, then centrifuged (Stratos, Thermo Fisher Scientific, Waltham, MA, USA) at 8000 rpm for 15 min. The supernatant was collected, the process repeated twice, and the extraction solutions combined. The merged supernatants were stored for the determination of the total phenolic, flavonoid, and flavanol contents, as well as the antioxidant activity and analysis of specific phenolic compounds.

### 2.5. Determination of Total Phenolic, Flavonoid, Flavanol, and Anthocyanin Contents

The measurement of the phenolic content was carried out using previously described methods [16]. The total phenolic content was quantified via the Folin–Ciocalteu method and expressed as milligrams of gallic acid equivalent (GAE) per 100 g of dry weight (DW) (mg GAE/100 g DW). The total flavonoid content was assessed by spectrophotometry using aluminum trichloride, as detailed by Dinçer et al. [17], with the results presented in mg rutin equivalents (RE) per 100 g of DW (mg RE/100 g DW). The flavanol content was determined using the *p*-dimethylaminocinnamaldehyde (*p*-DMACA)-hydrochloric acid method and expressed as milligrams of catechin equivalents (CE) per 100 g of DW (mg CE/100 g DW). The total anthocyanin content was evaluated using the pH differential method [18], with the results conveyed as milligrams of anthocyanins per 100 g of fresh weight (FW) (mg Cya3 glu/100 g FW). The total anthocyanin (TA) content was calculated using the following formula:

(1) $$TA=\frac{A\times V\times MW\times DF\times 100}{\varepsilon \times m}$$

*A* = (*A*_520nm_ − *A*_700nm_) pH_1.0_
*−* (*A*_520nm_ − *A*_700nm_) pH_4.5_ (2)

where *A* denotes the total absorbance of anthocyanins in the extraction solution, *A*_520nm_ denotes the absorbance of the sample measured at 520 nm, *A*_700nm_ denotes the absorbance of the sample measured at 700 nm, *V* denotes the volume of the extracting liquid (mL), *MW* denotes the relative molecular mass of cyanidin-3-glucoside at 449.2 g/mol, *DF* denotes the dilution factor of the samples, *ε* denotes the molar absorptivity of cyanidin-3-glucoside at 26,900, and *m* denotes the sample weight (g).

### 2.6. Determination of Antioxidant Capacity

(1) The DPPH (2,2-diphenyl-1-picrylhydrazyl) radical-scavenging assay was performed with slight modifications to the method described by Adiletta et al., 2016 [7]. An aliquot of 1 mL of the extract was mixed with 3.8 mL of a 0.12 g/mL DPPH solution in alcohol. The mixture was vigorously shaken and left in the dark for 30 min. Afterwards, the absorbance (*Ai*) was measured at 517 nm. For the blank control, absolute ethanol replaced the extract, and its absorbance (*Af*) was recorded. The percentage of DPPH radical-scavenging activity was calculated using the following equation:
  % Inhibition = (1 − *Ai*/*Af*) × 100% (3)
(2) The ABTS radical-scavenging assay was performed following the method of Re et al., 1999 [19], with modifications. To prepare the ABTS stock solution, 7 mmol/L of ABTS was mixed with 2.45 mmol/L of potassium persulfate and left to stand at room temperature for 12–16 h in the dark, forming the ABTS radical cation. This solution was then diluted with a 10 mmol/L phosphate buffer (pH 7.4) to obtain the ABTS test solution. For the assay, 3.9 mL of the ABTS test solution was combined with 0.1 mL of the extract, and the mixture was thoroughly mixed. The reaction was allowed to proceed in the dark at room temperature for 6 min before the absorbance was measured at 734 nm.
  *B* = (1 − *A*/0.7) × 100% (4)
  The rate of ABTS radical scavenging (*B*) was determined based on the absorbance (*A*) of the ABTS test solution with the seedless purple grape extract.
(3) The total peroxyl radical-trapping antioxidant parameter assay was adapted from Boumerfeg et al., 2009 [20]. A 0.1 mol/L sodium phosphate buffer (pH 7.4) was preheated to 37 °C. Then, 3 mL of this preheated buffer was transferred to a test tube, followed by the addition of 90 μL of a 5 mmol/L ABTS solution and 300 μL of a 200 μmol/L ABAP solution. The mixture was incubated at 37 °C for 5 min before the absorbance was promptly measured at 414 nm.

### 2.7. UPLC-VION-IMS-QTOF Analysis of Phenolic Compounds

The analysis of the phenolic compounds in raisins was conducted with modifications to the method described by Kolniak-Ostek J et al., 2015 [21]. The phenolic extract, prepared as detailed in Section 2.4, was utilized for analysis after filtration through a 2.2 µm filter membrane. Phenolic profiling was performed using UPLC-VION-IMS-QTOF (Waters Corporation, Milford, MA, USA). Chromatographic separation (Figure S5 in Supplementary Materials) was performed on a UPLC BEH C18 column (1.7 µm, 2.1 × 50 mm, Waters Corporation, Milford, MA, USA). The mobile phase comprised solvent A (0.1% formic acid in water) and solvent B (100% acetonitrile), with a gradient elution profile that was set as follows: 0–1 min, A/B (99:1, *v*/*v*); 1–12 min, A/B (99:1, *v*/*v*) to A/B (0:100, *v*/*v*); and 12–13.5 min, A/B (0:100, *v*/*v*) to A/B (99:1, *v*/*v*). The flow rate was 0.3 mL/min, the injection volume was 5 µL, and the column temperature was 45 °C. The ambient room temperature was maintained at 10 °C. The MS conditions included a capillary voltage of 2500 V, a cone voltage of 30 V, a source temperature of 100 °C, a desolvation temperature of 300 °C, and a desolvation gas flow (nitrogen) of 300 L/h. Compounds were identified by comparison with a self-built database containing 110 types of phenolic substances.

### 2.8. Statistical Analysis

Statistical analyses were performed using IBM SPSS Statistics Version 20 (SPSS Inc., Chicago, IL, USA). A one-way analysis of variance (ANOVA) was conducted to assess the results, and Duncan’s multiple range test was applied to identify significant differences among the raisin samples at a threshold of *p* < 0.05. Further analysis was conducted using SIMCA-P Version 14.1 (Umetrics, Umea, Sweden) for principal component analysis (PCA), orthogonal partial least squares–discriminant analysis (OPLS-DA), and the assessment of variable importance in projection (VIP).

## 3. Results

### 3.1. Effect of Different Pretreatment on the Quality of Raisins

The impact of various pretreatments on raisin quality is summarized in Table 1. The fructose and glucose contents were highest in the PT-treated raisins (25.65% and 25.2%, respectively), surpassing those in the DT and CK; the total and reducing sugar concentrations in the PT and DT were significantly greater than those in the CK (*p* < 0.05). The total acid content in the PT-treated raisins (31.15 g/kg) exceeded that in the CK (29.45 g/kg) and DT (28.75 g/kg) significantly (*p* < 0.05). The vitamin C (Vc) content was 0.115 mg/g for PT, 0.042 mg/g for DT, and 0.055 mg/g for CK, with notable differences observed among the pretreatment methods (*p* < 0.05). Amino acids such as aspartic acid, threonine, glutamic acid, alanine, valine, methionine, leucine, phenylalanine, lysine, and arginine were found in the highest concentrations in the PT compared to the CK and DT (*p* < 0.05). These results indicate that the physical pretreatment of raisins effectively preserves higher levels of nutrients.

### 3.2. Effect of Different Pretreatment on the Antioxidant Substances of Raisins

The influence of different pretreatments on the antioxidant components of raisins is detailed in Table 2. The total phenolic content in the PT was significantly higher, at 7.5 mg GAE/100 g of DW, compared to the DT and CK (*p* < 0.05). Similarly, the total flavonoid content was significantly greater in the PT-treated raisins (55.5 mg GAE/100 g DW) than in those from the CK (39.8 mg GAE/100 g DW) and DT (34.2 mg GAE/100 g DW) (*p* < 0.05). In contrast, the highest total flavanol content was observed in the CK (86.3 mg RE/100 g DW), followed by the PT (76.0 mg RE/100 g DW) and DT (33.7 mg RE/100 g DW), with this difference being statistically significant (*p* < 0.05). Moreover, the total anthocyanin content was highest in the PT (0.728 mg Cya3 glu/100 g FW), significantly surpassing the DT (0.534 mg Cya3 glu/100 g FW) and CK (0.288 mg Cya3 glu/100 g FW) (*p* < 0.05).

### 3.3. Effect of Different Pretreatment on the Antioxidant Capacity of Raisins

#### 3.3.1. DPPH and ABTS Radical-Scavenging

**Figure 2:**
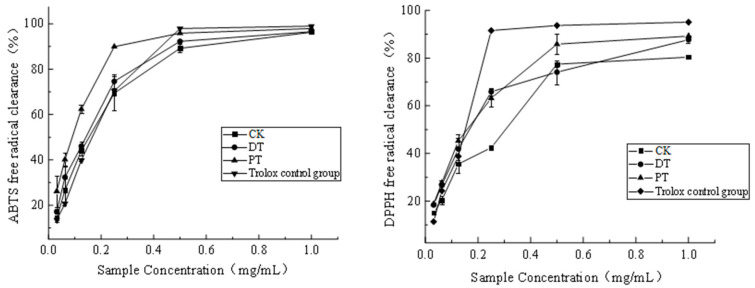
Different pretreatment methods for seedless purple grapes with ABTS and DPPH free radical clearance. Note: CK: the control group; DT: the drying agent treatment group; PT: the drying agent treatment group.

The DPPH and ABTS radical scavenging percentages are essential metrics for evaluating the antioxidant capacity [22]. As illustrated in Figure 2, the ABTS radical scavenging percentage increased with the sample concentration. Among all the concentrations tested, the PT exhibited the highest ABTS scavenging percentage, followed by the DT and CK, in that order. A similar pattern was observed for the DPPH radical scavenging activity, where the PT’s scavenging percentage exceeded that of the DT and CK at every sample concentration, except at 0.25 mg/mL. The IC_50_ values, representing the semi-inhibitory concentration, reflect the antioxidant capabilities of the samples; As illustrated in Table 3, the PT demonstrated the lowest IC_50_ value (0.091 mg/mL) for ABTS, indicating superior antioxidant activity, while the CK displayed the highest. The DPPH IC50 values aligned with the ABTS findings, highlighting that raisins treated physically possess the most robust antioxidant capacity.

#### 3.3.2. Total Radical Scavenging Antioxidant Capacity (TRAP)

**Figure 3:**
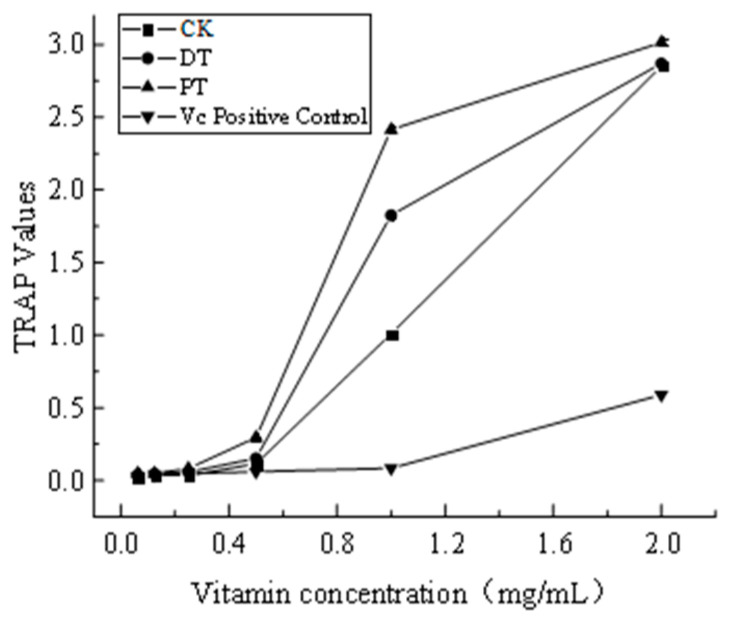
Effects of different pretreatment methods on the total antioxidant value of seedless purple raisins. Note: CK: the control group; DT: the drying agent treatment group; PT: the drying agent treatment group.

Figure 3 presents vitamin C as a positive control to assess the TRAP value of raisins following different pretreatments. The findings show that the TRAP value was significantly higher in the PT, with DT and CK decreasing in sequence. This trend aligns with the patterns observed in both the ABTS and DPPH radical scavenging activities.

### 3.4. Correlation between Antioxidant Substances and Antioxidant Capacity

**Figure 4:**
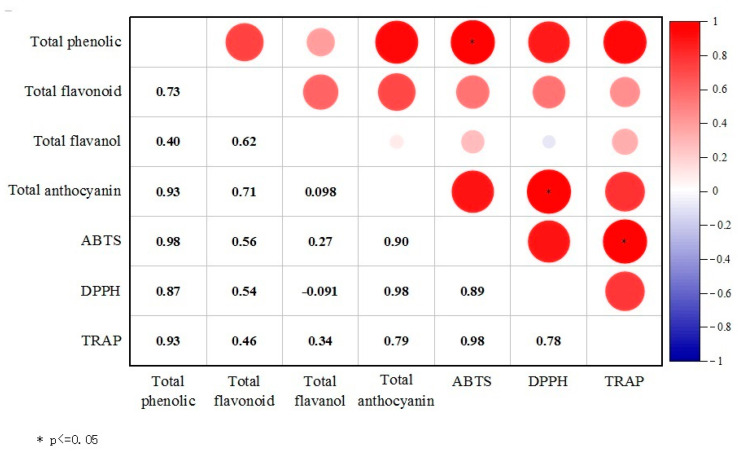
Correlation between antioxidant active substances and antioxidant capacity of seedless purple grapes under different pretreatments.

The correlation analysis between antioxidant substances and their scavenging capacities, as shown in Figure 4, identified significant associations. A notable correlation was found between the ABTS free radical scavenging rate and total phenolic content, and the correlation coefficient was 0.98 (*p* < 0.05). Similarly, a significant correlation existed between the total anthocyanin content and the DPPH free radical scavenging rate, while the correlation coefficient was also 0.98 (*p* < 0.05). Moreover, a positive correlation (*p* < 0.05) was observed between the ABTS scavenging rate and TRAP values. Except for a negative correlation between the total flavanol content and the DPPH scavenging rate (the correlation coefficient was −0.09), all other measured indices exhibited positive correlations.

### 3.5. Phenolic Substances

#### 3.5.1. Qualitative Analysis

Table 4 reveals that when using UPLC-VION-IMS-QTOF, a total of 35 phenolic compounds and 4 flavan-3-ols were detected in seedless purple grapes. Epigallocatechin gallate, with a response value of 1860, was uniquely identified in the DT group. The PT displayed significantly higher levels of the rest of the phenolic compounds compared to the CK and DT (*p* < 0.05). Sixteen phenolic acids were found, with the CK, DT, and PT containing 12, 14, and 15 compounds, respectively. Homovanillic acid (response value 5092) was exclusively detected in the PT, while caffeic acid (response value 1352) was unique to the CK. The PT’s concentrations of chlorogenic acid, ethyl coumarate, 3,5-O-dicaffeoylquinic acid, homovanillic acid, cinnamic acid, sinapic acid, and syringic acid were significantly higher (*p* < 0.05) than those in the CK and DT. Among the fourteen flavonols identified, Kaempferide was missing only in the DT. Naringenin and Galangin were not found in either the DT and PT, and dihydrokaempferide was absent in the CK. Dihydroquercetin-3-O-rhamnoside was exclusively present in the DT. Moreover, the PT’s levels of dihydrokaempferide (response value 4207), Morin (response value 119,447), and Phlorizin (response value 6652) were significantly higher (*p* < 0.05) than in the other groups. Resveratrol, among the stilbenoid compounds, was identified in the PT group and exhibited a response value of 16,544, markedly higher than that of the CK and DT.

#### 3.5.2. Heat Map of Phenols

**Figure 5:**
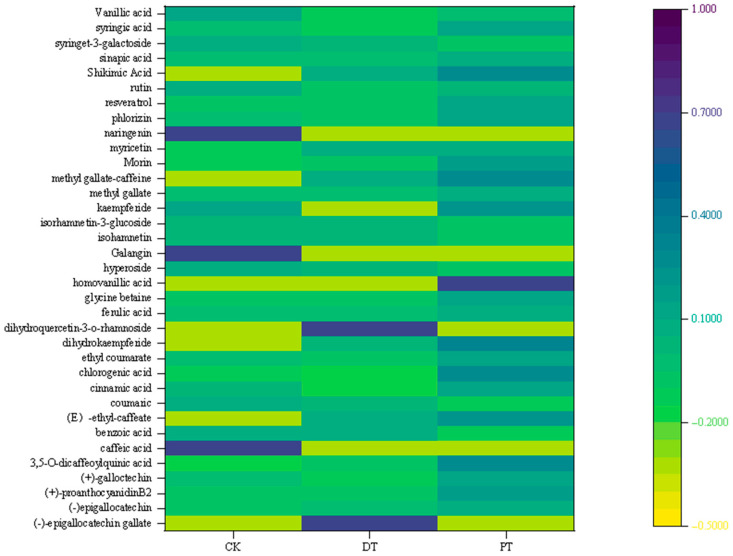
Heat map of phenolic substances in seedless purple grapes under different pretreatment methods.

The study employed heatmaps to visually represent the qualitative response values of phenolic compounds in seedless purple grapes, as depicted in Figure 5. Darker blue hues indicate a higher content of each phenolic substance among the groups, while darker orange hues suggest a lower content. The PT group displayed predominantly blue hues, indicating a higher quantity of most phenolic compounds compared to the other groups.

Among the 35 phenolic compounds identified, substances like (E)-ethyl caffeate, dihydrokaempferide, methyl gallate-caffeine, and shikimic acid were found in the seedless raisins processed by both the DT and PT methods, with slightly higher levels in the PT. Homovanillic acid was exclusively identified in the PT. A total of 21 compounds were most abundant in the PT. Conversely, four compounds, including epigallocatechin gallate and dihydroquercetin-3-O-rhamnoside (both unique to DT), myricetin, and isorhamnetin, showed the highest concentrations in the DT group.

The CK exhibited the highest levels of 10 compounds. Notably, caffeic acid, naringenin, and galangin were exclusively found in the CK, highlighted by dark blue in the heatmap. Additionally, the CK had the highest level of syringin-3-galactoside, while the concentrations of the remaining compounds were marginally higher than those in the DT or PT, without significant differences.

### 3.6. OPLS-DA Modeling for Raisins with Different Pretreatments

**Figure 6:**
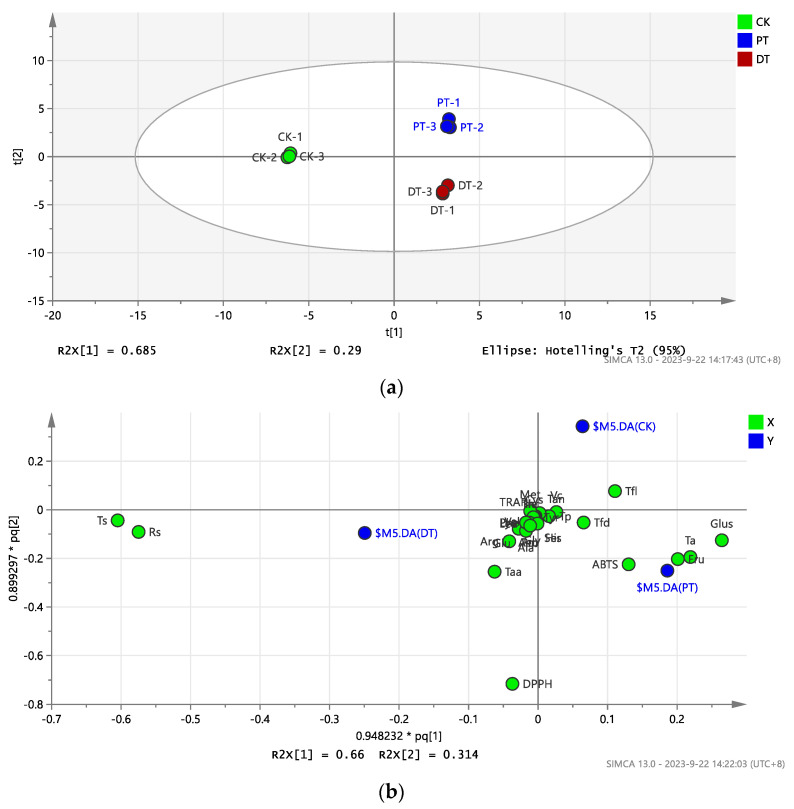

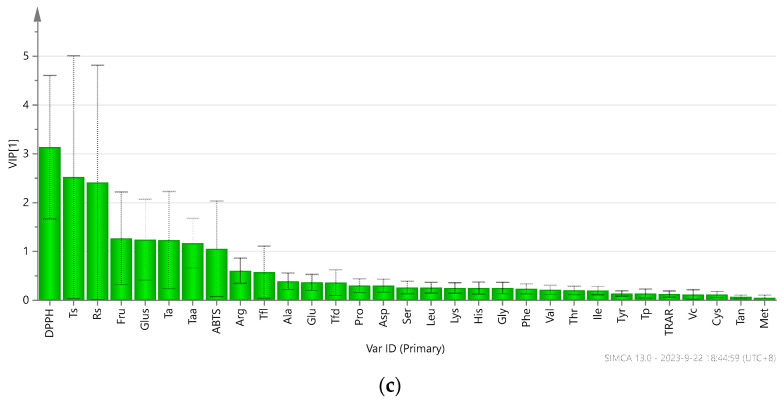
OPLS-DA modeling of the raisins with different pretreatments: (**a**) PCA score; (**b**) score plots of raisin samples; (**c**) VIP plots of variables. DPPH = 2,2-diphenyl-1-picrylhydrazyl; Ts = Total sugar; Rs = Reducing sugar; Glus = Glucose; Ta = Total acid; Fru = Fructose; ABTS = 2,2′-azino-bis(3-ethylbenzothiazoline-6-sulfonic acid); Taa = Total amino; Tfl = Total flavanol; Arg = Arginine; Tfd = Total flavonoid; Ala = Alanine; Glu = Glutamic acid; Asp = Aspartic acid; Pro = Proline; Ser = Serine; Leu = Leucine; Lys = Lysine; Gly = Glycine; His = Histidine; Phe = Phenylalanine; Val = Valine; Thr = Threonine; Ile = Isoleucine; Tp = Total phenolic; Vc = Vitamin C; Tyr = Tyrosine; TRAR = Total radical scavenging antioxidant capacity; Cys = Cysteine; Tan = Total anthocyanin; Met = Methionine.

OPLS-DA models were developed to distinguish between the raisins treated by the three different methods. The analysis, based on 31 indices for 9 raisin samples, is depicted in Figure 6. PCA analysis showed clear differentiation among the three groups of raisins, with each subjected to a unique treatment (Figure 6a). The loading distribution of variables indicated that fructose, total acid, glucose, and ABTS were characteristic of the PT-treated raisins, while reducing sugar, total sugar, DPPH, and total amino acids were more indicative of the DT-treated raisins. The flavanol content was a distinguishing feature of the CK-treated raisins (Figure 6b). The VIP plot highlighted that the DPPH, total sugar, reducing sugar, glucose, total acid, fructose, ABTS, and total amino acids were the most discriminant variables, each with a VIP score above 1 (Figure 6c).

## 4. Discussion

The consumption of fruits plays a vital role in health and well-being. Seedless purple raisins, abundant in various nutritional compounds, contribute to preventing a range of diseases [23]. Due to the limited shelf life of fresh grapes, many are transformed into raisins to extend their shelf life [7,10]. Traditionally, raisins are produced through sun drying, which significantly reduces their moisture content over 8–10 days [6]. Studies have shown that the duration of grape dehydration influences their polyphenol content, ascorbic acid content, and antioxidant activity [3]. Therefore, minimizing drying times is essential for improving raisin quality. The challenge in drying grapes is primarily due to their waxy outer layer, prompting the exploration of various pretreatment methods to eliminate this barrier. Therefore, pretreating grapes to remove the wax on their surface before making raisins is very important. The pretreatment used before drying raisins includes physical pretreatment and chemical pretreatment, and the nutritional value and antioxidant activity of raisins can be substantially affected by the pretreatment methods applied to the grapes [3].

### 4.1. Effect of Pretreatment and Drying on the Nutritional Quality of Raisins

Pretreatment not only accelerates fruit drying times but also improves the nutritional quality of fruit. Grapes treated with olive oil have a drying time that is reduced to 22.5 h from 41 h for untreated grapes, achieving a 43% time saving [3]; a 50% reduction in the e drying time was also achieved by treating Thompson seedless grapes with a mix of 5% K_2_CO_3_ and 2% ethyl oleate at 60 °C [24]. A solution of 0.5% olive oil and 6% potassium carbonate decreased the drying time compared to a 2.5% olive oil pretreatment [25]. Like a 50 s cold plasma pretreatment, it significantly increased the moisture diffusivity, reducing the drying time by up to 26.27% [5]. Pulsed electric field (PEF) and ultrasound (US) treatments have significantly reduced the drying times for orange peels and kiwifruit while improving or preserving the product quality, including the color and ascorbic acid retention, among others. Because PEF pretreatments can affect the product structure and the influence of US depends on the internal structure of products, the combination of both techniques could have a synergistic effect [26,27]. Electromagnetic radiation (EMR) pretreatment, combined with low-humidity air drying, has decreased the drying time for apple slices by 23.4 to 27.3%, with EMR-treated slices exhibiting higher ascorbic acid retention, a higher total phenolic content and antioxidant activity, and better color retention [28]. Jun-Wen Bai et al. [29] investigated the effects of hot-air impingement blanching (HHAIB) on raisin quality, finding that this non-chemical pretreatment not only sped up the drying kinetics but also improved the color parameters of seedless grapes.

In this study, pretreatment is applied to fruit drying, the drying efficiency is improved, and the nutritional quality of the fruit is promoted or maintained. But research on the nutritional quality of fruits dried using such pretreatments is lacking and incomplete. In this study, the effect of pretreatment on the drying efficiency and nutritional quality of raisins was comprehensively evaluated. It was found that both chemical and physical pretreatments are effective in shortening the drying times of grapes. Under the same drying conditions, the physical pretreatment reduced the drying time by 29.63% compared to the chemical pretreatment, and by 48.65% compared to the untreated grapes, making physical pretreatment the most efficient method for drying grapes. These findings are consistent with the results of other studies. Additionally, in this study, physically pretreated raisins had higher fructose and glucose contents compared to chemically treated and untreated raisins. Although chemically treated raisins had the highest total and reducing sugar contents, the differences were not significant when compared to physically pretreated raisins. The vitamin C content was the lowest in chemically pretreated raisins. The amino acid content was significantly higher in all pretreated raisins compared to the untreated ones. Overall, raisins subjected to the physical treatment demonstrated superior nutritional quality, followed by those treated chemically.

### 4.2. Effect of Pretreatment and Drying on the Functional Quality of Raisins

Grapes contain abundant bioactive substances, especially polyphenols, which have excellent functional qualities. Raisins rank among the highest in their concentration of total phenolic compounds and have the highest levels of total antioxidant activity among solid fruit products [30,31]. In this study, UPLC-VION-IMS-QTOF pinpointed 35 distinct phenolic compounds, with flavonols and phenolic acids being predominant. Importantly, the content of resveratrol, a key stilbene found mainly in grape skins and mostly in the trans form, was significantly elevated in grapes treated physically [32]. Various research has shown significant variances in the total phenolic content and antioxidant activities of raisins according to the pretreatment applied. For example, NaOH pretreatment led to a reduction in the ascorbic acid content, mineral content and antioxidant activity of raisins, while microwave-assisted hot-air drying increased their antioxidant activity [6,9]; meanwhile, treating raisins with olive oil before production changes their nutritional composition, with treated raisins retaining more anthocyanins and proanthocyanidins than untreated ones [3]. An investigation into the drying of flame seedless raisins using five different pretreatment methods discovered that the drying duration for grapes pretreated with dipping was less than half of that for control grapes. Ultrasound pretreatment efficiently preserved the total phenolic content, though it impacted the total flavonoid content, while enzymatic pretreatment had a minimal effect on the total flavonoid content; in the radical scavenging antioxidant capacity assay, slight differences were observed between the pretreatments [33]. Moreover, emerging drying pretreatment technologies, such as cold plasma pretreatment, have shown potential. This technique has improved the quality of dried grapes, increasing the total phenolic content, antioxidant activity, and vitamin C retention by 3.06–30.53%, 7.31–62.29%, and 17.87–168.73%, respectively, compared to untreated grapes [5].

In summary, the authors found that different pretreatment methods have different effects on the functional quality of raisins. Chemical pretreatment methods may affect the functional quality of raisins, while physical pretreatment has a maintaining or promoting effect on the functional quality of raisins. The physical pretreatment method used in this study can better increase the content of total phenolics, total flavonoids, and total anthocyanins in raisins, and enhance their capacity for ABTS and DPPH radical scavenging, which has a good promoting effect on the functional quality of raisins. Compared with microwave pretreatment, the physical pretreatment we adopted has a better effect on improving the functional quality of raisins. Compared with cold plasma pretreatment, the equipment designed in this experiment is simpler and more convenient. Therefore, the physical pretreatment adopted in this experiment has bright prospects.

## 5. Conclusions

This study evaluated the effects of different pretreatment methods on the quality of raisins subjected to uniform hot-air drying conditions. The assessment focused on the nutritional and functional quality of raisins produced with seedless purple grapes. The findings indicated that the physical pretreatment significantly elevated the levels of vitamin C, fructose, glucose, total acid, total phenolics, total flavonoids, total anthocyanins, and other antioxidative parameters, surpassing both the chemical pretreatment and the untreated control group. Notably, the concentrations of key phenolic compounds such as resveratrol, ferulic acid, chlorogenic acid, ethyl coumarate, and cinnamic acid were considerably higher in the physically pretreated raisins. These results demonstrate that raisins resulting from physical pretreatment methods possess a superior nutritional quality. Moreover, physical pretreatment, as a non-chemical approach, does not alter the grapes’ inherent nature and avoids the presence of chemical residues in the final product. Looking ahead, the equipment used for the physical pretreatment of grapes could be further refined to facilitate its application in large-scale production.

## Figures and Tables

**Table 1:** Comparison of nutritional quality of raisins after different pretreatments.

Nutritional Quality Index	Different Pretreatments		
	CK	DT	PT
Fructose (%)	23.8 ± 0.283 ^b^	23.45 ± 0.071 ^b^	25.65 ± 0.212 ^a^
Glucose (%)	23.6 ± 0.141 ^b^	22.5 ± 0.134 ^b^	25.2 ± 0.283 ^a^
Total sugar (%)	59.2 ± 1.414 ^b^	68.7 ± 1.131 ^a^	67.3 ± 1.273 ^a^
Reducing sugar (%)	57.75 ± 1.061 ^b^	66.95 ± 1.626 ^a^	66.65 ± 0.071 ^a^
Total acid (g/kg)	29.45 ± 1.202 ^b^	28.75 ± 0.212 ^c^	31.15 ± 0.212 ^a^
Vc (mg/g)	0.055 ± 0.003 ^b^	0.042 ± 0.005 ^b^	0.115 ± 0.005 ^a^
Aspartic acid (g/100 g)	0.061 ± 0.001 ^c^	0.135 ± 0.001 ^b^	0.139 ± 0.001 ^a^
Threonine (g/100 g)	0.031 ± 0.001 ^c^	0.066 ± 0.001 ^b^	0.069 ± 0.001 ^a^
Serine (g/100 g)	0.041 ± 0.001 ^c^	0.102 ± 0.00 ^a^	0.089 ± 0.001 ^b^
Glutamic acid (g/100 g)	0.113 ± 0.001 ^c^	0.218 ± 0.002 ^b^	0.248 ± 0.001 ^a^
Glycine (g/100 g)	0.042 ± 0.001 ^c^	0.095 ± 0.002 ^a^	0.093 ± 0.001 ^b^
Alanine (g/100 g)	0.101 ± 0.001 ^c^	0.227 ± 0.001 ^b^	0.231 ± 0.001 ^a^
Cysteine (g/100 g)	0.012 ± 0.001 ^b^	0.024 ± 0.001 ^a^	0.024 ± 0.001 ^a^
Valine (g/100 g)	0.038 ± 0.001 ^c^	0.076 ± 0.001 ^b^	0.080 ± 0.001 ^a^
Methionine (g/100 g)	0.012 ± 0.001 ^b^	0.012 ± 0.00 ^b^	0.016 ± 0.00 ^a^
Isoleucine (g/100 g)	0.029 ± 0.001 ^b^	0.063 ± 0.001 ^a^	0.064 ± 0.001 ^a^
Leucine (g/100 g)	0.048 ± 0.001 ^c^	0.103 ± 0.000 ^b^	0.108 ± 0.001 ^a^
Tyrosine (g/100 g)	0.011 ± 0.001 ^b^	0.027 ± 0.001 ^a^	0.028 ± 0.001 ^a^
Phenylalanine (g/100 g)	0.040 ± 0.001 ^c^	0.085 ± 0.001 ^b^	0.089 ± 0.001 ^a^
Histidine (g/100 g)	0.046 ± 0.001 ^c^	0.103 ± 0.001 ^a^	0.089 ± 0.001 ^b^
Lysine (g/100 g)	0.049 ± 0.001 ^c^	0.099 ± 0.00 ^b^	0.109 ± 0.001 ^a^
Arginine (g/100 g)	0.241 ± 0.001 ^c^	0.534 ± 0.001 ^b^	0.592 ± 0.003 ^a^
Proline (g/100 g)	0.064 ± 0.001 ^c^	0.142 ± 0.001 ^a^	0.138 ± 0.009 ^b^

Note: In the table, different lowercase letters within the same column denote a significant difference at *p* < 0.05. CK: the control group; DT: the drying agent treatment group; PT: the drying agent treatment group.

**Table 2:** Comparison of antioxidant substances of raisins after different pretreatments.

Different Pretreatments	Total Phenolic (mg GAE/100 g DW)	Total Flavonoid (mg RE/100 g DW)	Total Flavanol (mg RE/100 g DW)	Total Anthocyanin (mg Cya3 glu/100 g FW)
CK	5.5 ± 0.0025 ^b^	39.8 ± 0.018 ^b^	86.3 ± 0.017 ^a^	0.288 ± 0.007 ^c^
DT	5.7 ± 0.0025 ^b^	34.2 ± 0.014 ^b^	33.7 ± 0.005 ^c^	0.534 ± 0.004 ^b^
PT	7.5 ± 0.0019 ^a^	55.5 ± 0.014 ^a^	76.0 ± 0.015 ^b^	0.728 ± 0.006 ^a^

Note: In the table, different lowercase letters within the same column denote a significant difference at *p* < 0.05. CK: the control group; DT: the drying agent treatment group; PT: the drying agent treatment group.

**Table 3:** IC_50_ values for antioxidant activity of seedless purple grapes with different pretreatment methods.

	CK	DT	PT
Scavenge ABTS radical IC_50_ (mg/mL)	0.151 ^a^	0.132 ^a^	0.091 ^b^
Scavenge DPPH radical IC_50_ (mg/mL)	0.292 ^a^	0.186 ^b^	0.131 ^b^

Note: In the table, different lowercase letters within the same column denote a significant difference at *p* < 0.05. CK: the control group; DT: the drying agent treatment group; PT: the drying agent treatment group.

**Table 4:** Qualitative analysis of phenolic compounds in seedless purple grapes subjected to various pretreatment methods.

Classification	Phenolic Substance	CK	DT	PT
Flavan-3-ol	(-)-Epigallocatechin gallate	ND	1860 ± 421 ^a^	ND
	(-)-Epigallocatechin	19,407 ± 1182 ^c^	23,611 ± 795 ^b^	32,289 ± 555 ^a^
	(+)-ProanthocyanidinB_2_	3593 ± 153 ^b^	3332 ± 79.16 ^b^	7085 ± 778 ^a^
	(+)-Galloctechin	2679 ± 164 ^b^	2014 ± 39.61 ^b^	4036 ± 258 ^a^
Phenolic acids	Ferulic acid	3017 ± 149 ^a^	2993 ± 232 ^a^	4066 ± 260 ^a^
	Vanillic acid	2981 ± 807 ^a^	1437 ± 164 ^a^	2076 ± 89 ^a^
	Caffeic acid	1352 ± 127 ^a^	ND	ND
	(E)-Ethyl caffeate	ND	1406 ± 149 ^a^	1947 ± 270 ^a^
	Coumaric	14,002 ± 386 ^a^	13,406 ± 580 ^a^	8248 ± 262 ^b^
	Chlorogenic acid	2871 ± 81 ^b^	2431 ± 536 ^b^	8149 ± 1081 ^a^
	Ethyl coumarate	13,925 ± 532 ^b^	12,735 ± 466 ^b^	20,739 ± 235 ^a^
	Methyl gallate	1323 ± 57 ^a^	1209 ± 16.81 ^a^	1715 ± 58 ^a^
	Methyl gallate-caffeine	ND	1862 ± 41.8 ^a^	2761 ± 167 ^a^
	3,5-O-dicaffeoylquinic acid	9279 ± 379 ^b^	12,885 ± 529 ^b^	32,698 ± 853 ^a^
	Homovanillic acid	ND	ND	5092 ± 394 ^a^
	Cinnamic acid	69,563 ± 2310 ^b^	31,739 ± 456 ^c^	93,704 ± 1839 ^a^
	Benzoic acid	4108 ± 191 ^a^	4056 ± 122 ^a^	1877 ± 41 ^b^
	Shikimic Acid	ND	1225 ± 21 ^a^	1805 ± 252 ^a^
	Sinapic acid	18,195 ± 1657 ^b^	17,470 ± 2153 ^b^	25,869 ± 8250 ^a^
	Syringic acid	14,311 ± 1296 ^b^	8703 ± 1385 ^c^	20,469 ± 606 ^a^
Flavonols	Dihydrokaempferide	ND	2296 ± 169 ^b^	4207 ± 592 ^a^
	Dihydroquercetin-3-o-rhamnoside	ND	1268 ± 56.4 ^a^	ND
	Hyperoside	13,866 ± 783 ^a^	12,914 ± 289 ^a^	8628 ± 253 ^b^
	Isohamnetin	34,812 ± 1107 ^a^	37,719 ± 216 ^a^	26,762 ± 621 ^b^
	Isorhamnetin-3-glucoside	13,019 ± 603 ^a^	12,614 ± 458.6 ^a^	8896 ± 420 ^b^
	Galangin	1321 ± 62 ^a^	ND	ND
	Morin	50,490 ± 4038 ^b^	55,914 ± 1846.7 ^b^	119,447 ± 1575 ^a^
	Myricetin	1792 ± 280 ^b^	3581 ± 173 ^a^	3517 ± 188 ^a^
	Phlorizin	4480 ± 177 ^b^	3843 ± 560 ^b^	6652 ± 24 ^a^
	Kaempferide	1571 ± 69 ^a^	ND	1873 ± 13 ^a^
	Naringenin	1234 ± 73 ^a^	ND	ND
	Rutin	7784 ± 278 ^a^	5189 ± 52.9 ^b^	7231 ± 519 ^a^
	Syringet-3-galactoside	19,502 ± 806 ^a^	16,814 ± 549 ^b^	13,521 ± 581 ^b^
	Glycine betaine	2217 ± 137 ^a^	2150 ± 20 ^a^	3563 ± 81 ^a^
Stilbenes	Resveratrol	8798 ± 137 ^b^	8993 ± 391 ^b^	16,544 ± 440 ^a^

Note: In the table, different lowercase letters within the same column denote a significant difference at *p* < 0.05. ND indicates not detected. CK: the control group; DT: the drying agent treatment group; PT: the drying agent treatment group.

## Data Availability

The original contributions presented in the study are included in the article/Supplementary Materials, further inquiries can be directed to the corresponding author.

## Supplementary Materials

The following supporting information can be downloaded at: https://www.mdpi.com/article/10.3390/foods13081138/s1, Figure S1: The standard chromatogram of fructose, glucose, and sucrose; Figure S2: Detection of fructose, glucose, and sucrose profiles in the sample; Figure S3: The red line represents the spectrum of amino acid standards at 570 nm, while the blue line represents the spectrum of amino acid standards at 440 nm; Figure S4: The red line represents the spectrum of amino acids in the sample at 570 nm, while the blue line represents the spectrum of amino acids in the sample at 440 nm; Figure S5: Total ion chromatogram of phenolic substances in raisins based on UPLC-VION-IMS-QTOF.
