# Supplementary material for: Effect of Pretreatment and Drying on the Nutritional and Functional Quality of Raisins Produced with Seedless Purple Grapes
Source: Foods. 2024 Apr 9;13(8):1138. doi: 10.3390/foods13081138 (PMC11048869; doi:10.3390/foods13081138)
Supplement: Supplementary file 1 [file foods-13-01138-s001.zip › foods-2919595-supplementary.pdf]

Supplementary materials

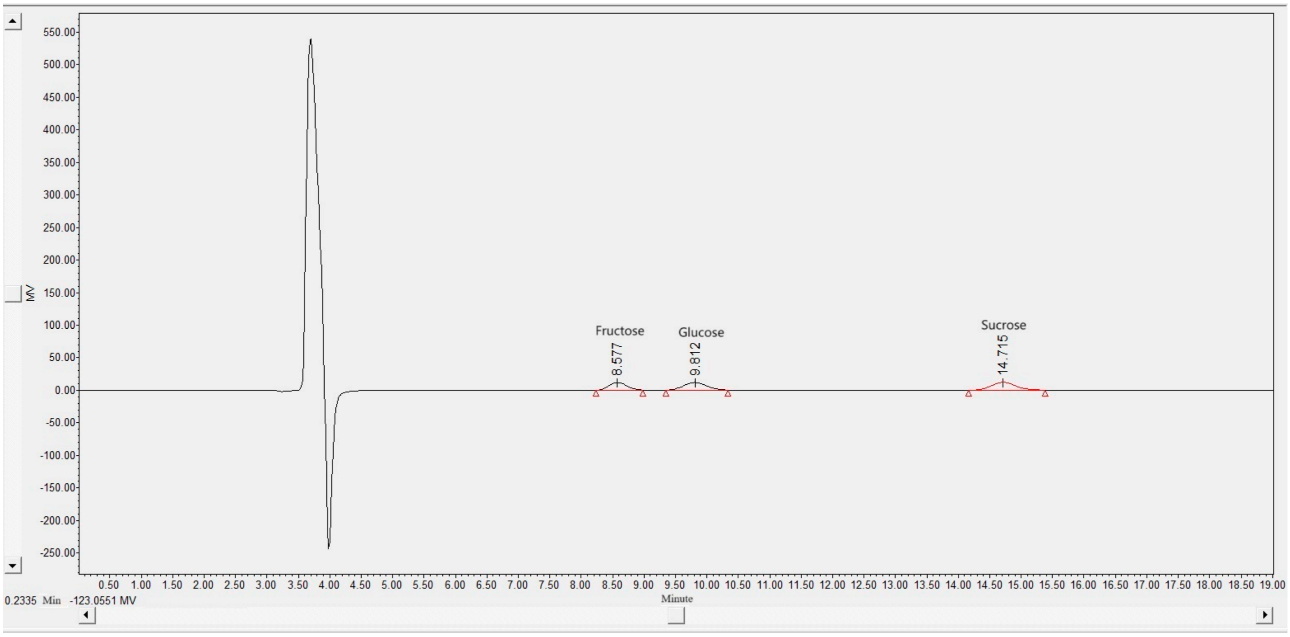

Figure S1：The standard chromatogram of fructose, glucose, and sucrose

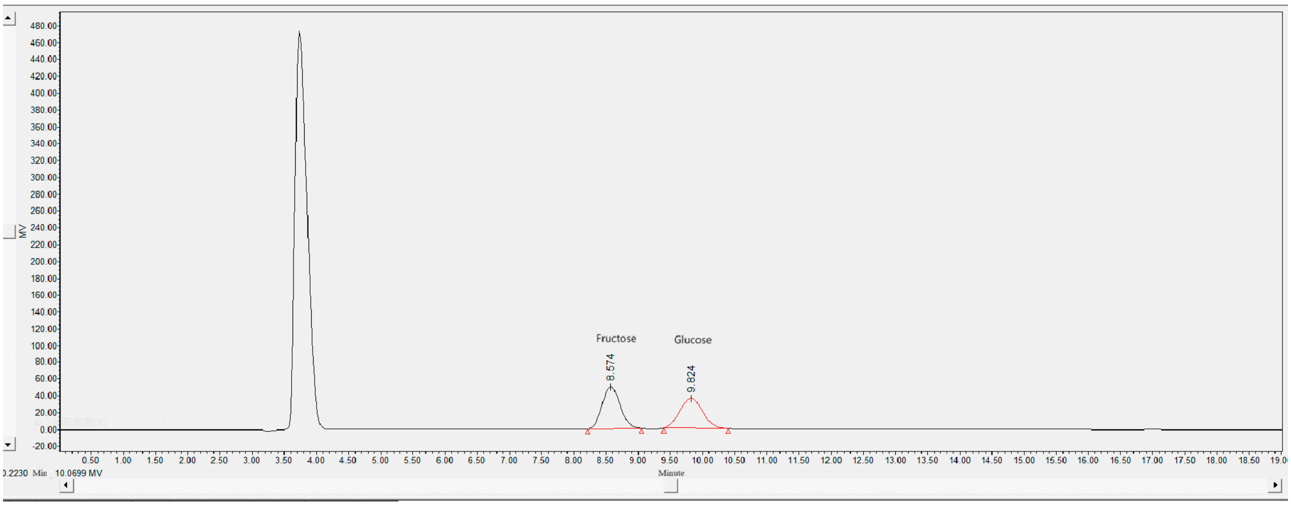

Figure S2：Detection of fructose, glucose, and sucrose profiles in the sample

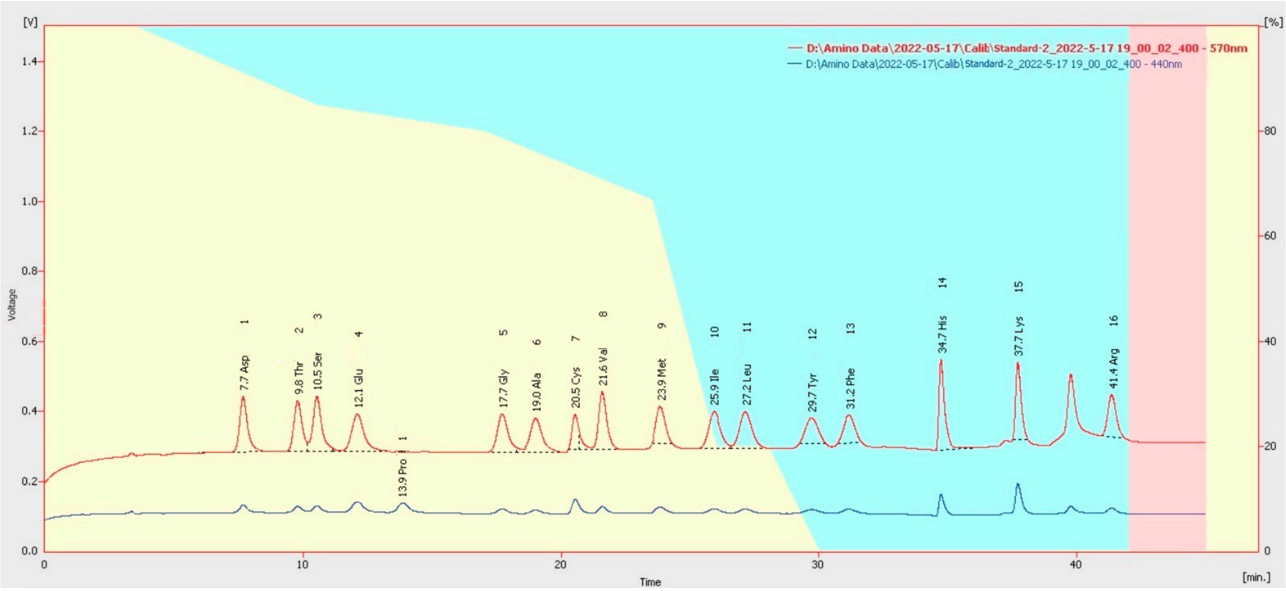

Figure S3：The red line represents the spectrum of amino acid standards at 570 nm, while the blue line represents the spectrum of amino acid standards at 440 nm. Note: Asp = Aspartic acid; Thr = Threonine; Ser = Serine; Glu = Glutamic acid; Pro = Proline; Gly = Glycine; Ala = Alanine; Cys = Cysteine; Val = Valine; Met = Methionine; Ile = Isoleucine; Leu = Leucine; Tyr = Tyrosine; Phe = Phenylalanine; His = Histidine; Lys = Lysine; Arg = Arginine

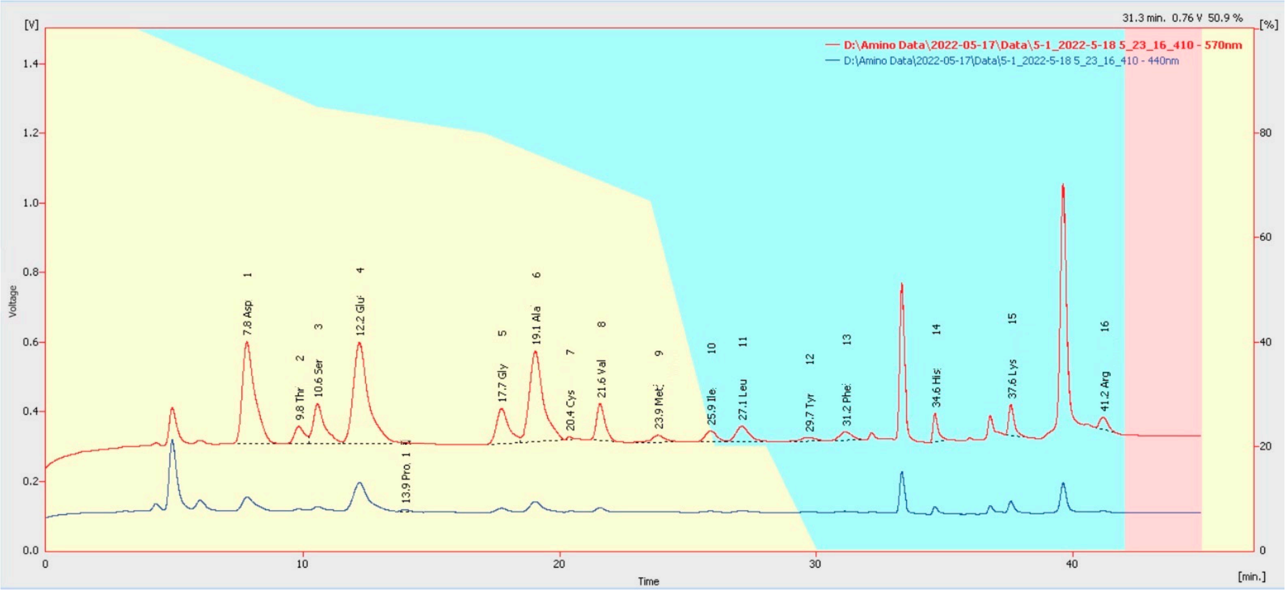

Figure S4：The red line represents the spectrum of amino acids in the sample at 570 nm, while the blue line represents the spectrum of amino acids in the sample at 440 nm. Note: Asp = Aspartic acid; Thr = Threonine; Ser = Serine; Glu = Glutamic acid; Pro = Proline; Gly = Glycine; Ala = Alanine; Cys = Cysteine; Val = Valine; Met = Methionine; Ile = Isoleucine; Leu = Leucine; Tyr = Tyrosine; Phe = Phenylalanine; His = Histidine; Lys = Lysine; Arg = Arginine

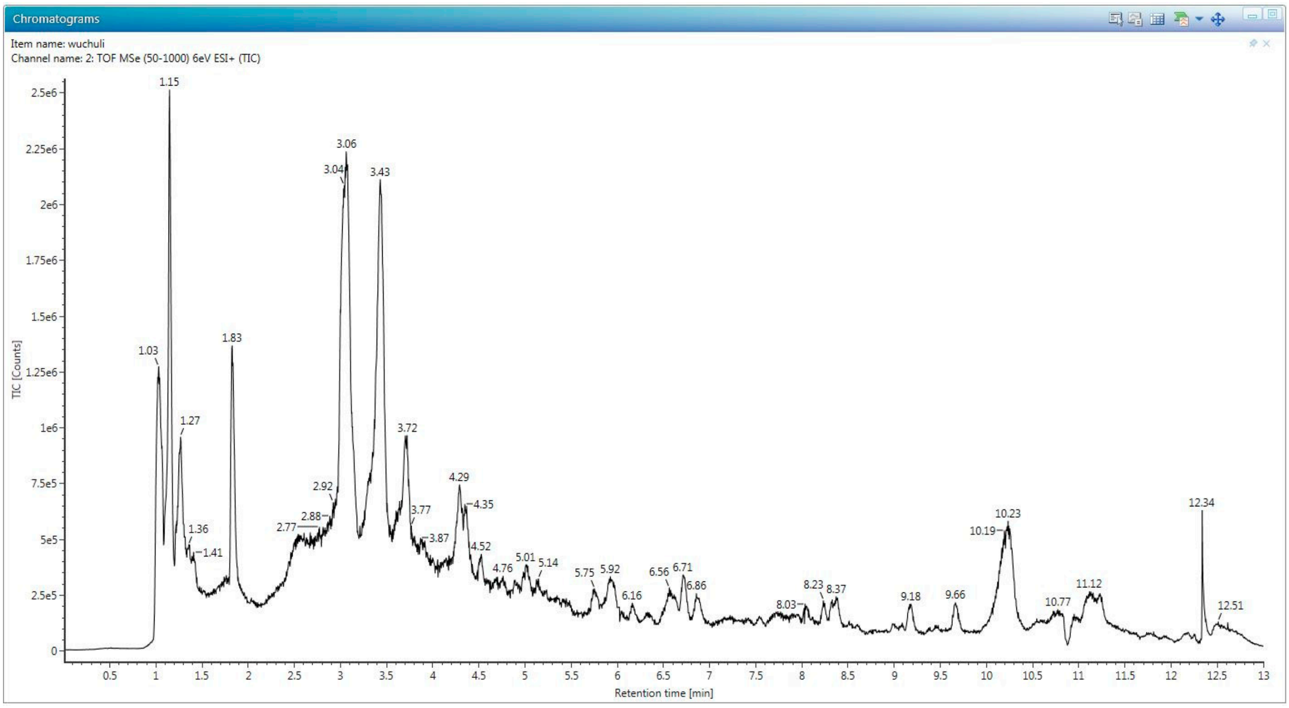

Figure S5：Total ion chromatogram of phenolic substances in raisins based on UPLC-VION-IMS-QTOF
